# All-Solid-State Carbon Black Paste Electrodes Modified by Poly(3-octylthiophene-2,5-diyl) and Transition Metal Oxides for Determination of Nitrate Ions

**DOI:** 10.3390/molecules28114313

**Published:** 2023-05-24

**Authors:** Barbara Niemiec, Robert Piech, Beata Paczosa-Bator

**Affiliations:** Faculty of Materials Science and Ceramics, AGH University of Science and Technology, Mickiewicza 30, PL-30059 Krakow, Poland

**Keywords:** nitrate-selective electrodes, paste electrodes, carbon black, poly(3-octylthiophene-2,5-diyl), potentiometric sensors

## Abstract

This paper presents new paste ion-selective electrodes for the determination of nitrate ions in soil. The pastes used in the construction of the electrodes are based on carbon black doped with transition metal oxides: ruthenium, iridium, and polymer-poly(3-octylthiophene-2,5-diyl). The proposed pastes were electrically characterized by chronopotentiometry and broadly characterized potentiometrically. The tests showed that the metal admixtures used increased the electric capacitance of the pastes to 470 μF for the ruthenium-doped paste. The polymer additive used positively affects the stability of the electrode response. All tested electrodes were characterized by a sensitivity close to that of the Nernst equation. In addition, the proposed electrodes have a measurement range of 10^−5^ to 10^−1^ M NO_3_^−^ ions. They are impervious to light conditions and pH changes in the range of 2–10. The utility of the electrodes presented in this work was demonstrated during measurements directly in soil samples. The electrodes presented in this paper show satisfactory metrological parameters and can be successfully used for determinations in real samples.

## 1. Introduction

The role of nitrate ions in plant development is essential because they serve as a crucial source of nitrogen for plant growth and metabolism. Nitrate ions are taken up by plant roots from the soil and then transported to various plant tissues, where they are converted into amino acids and other important molecules. Research has shown that nitrate ions also play a significant role in regulating plant growth and development, including root and shoot growth, branching, and flowering. In addition, they have been found to influence the expression of genes involved in plant development and stress responses [1,2]. In general, the role of nitrate ions in plant development is complex and multifaceted, involving a range of biochemical and physiological processes. The importance of this essential nutrient in plant growth and development cannot be overstated.

Knowing the content of nitrate ions in the soil allows the adjustment of the fertilizer used and the fertilization schedule. Therefore, it is important to control the content of biophilic ions in the cultivated soil. It also allows the avoidance of the phenomenon of overfertilization of the soil, which has a negative impact on both the development of plants and the surrounding environment. For this reason, it is necessary to keep the concentration of nitrate ions in the optimum range.

Potentiometric measurements are one of the most widely used measurement techniques. They are popular due to the simplicity of the technique, short measurement time, ease of construction, and a wide linear range. Furthermore, the technique is nondestructive to the samples and can be used in the presence of matrix components. In addition, the technique requires low consumption of chemicals and can be used to determine the concentration of the analyte in a small sample amount. In addition, all-solid-state sensors can be used for in situ measurements. Unlike conventional electrodes, they do not have an internal solution in their construction, which allows for further miniaturization of the electrodes, allows the possibility of keeping them in any position, and eliminates the problem of internal solution leakage into the sample and the need to replenish it [3].

The simplest construction of all-solid-state electrodes is a coated wire/disc electrode [4]. In this type of electrode, an ion-selective membrane (ISM) directly covers the substrate of the electrode, for example, a carbon glass disc. This solution has many drawbacks caused by the direct connection of two types of conductors, ionic and electronic. To solve this problem, another layer can be placed between ISM and the substrate of the electrode. This layer, called the solid-contact layer, is characterized by mixed types of conductivity, ionic and electronic. This type of electrode is called a solid contact electrode. This solution improves most of the electrode parameters, such as potential stability, reversibility of response, or electric capacity.

So far, many materials have been tested as intermediate layers of electrodes, including: many carbon materials, such as carbon black (CB) [5], single and multi-walled carbon nanotubes [6,7,8], or chemically reduced graphene oxide [9,10,11]; some transition metal oxides, such as hydrous ruthenium [12,13,14] and iridium dioxides [15]; conducting polymers, including poly(3-octylthiophene) [16,17] and polypyrrole [18]; and organic crystals, such as 7,7,8,8-tetracyanoquinodimethane [19] and tetrathiafulvalene (TTF) [20].

In most solid-contact electrode constructions, the intermediate layer is dropped or deposited on the surface of the substrate electrode. Paste electrodes are another type of this electrode. In this solution, a paste of components mixed with paraffin oil is placed in the cavity of the electrode [21,22,23,24]. The leveled surface of the paste is already a modified electrode substrate. This solution allows the reduction of the consumption of harmful solvents such as DMF or THF. These solvents are often used in the preparation of intermediate layer solution. Reducing the use of solvents, ease of preparation and the simplicity of construction are the clear advantages of paste electrodes. In addition, paste electrodes are reusable electrodes. It is sufficient to remove the ISM, push out a little paste and smooth its surface. After such surface renewal, the electrode is again ready to be covered with an ISM. The paste used in paste electrodes should, like the intermediate layers in solid-contact electrodes, meet certain requirements. These include: providing a quick and reversible transition from ionic to electronic conductivity; and having a non-polarizable surface between the paste and the high-capacity red-ox or double-layer ISM. In addition, the paste should be hydrophobic and characterized by low water absorption, which prevents the formation of a water layer. Moreover, the materials for the paste should be readily available, economical, and non-toxic.

Previous investigations by Lenar et al. [14,15,25] have shown that hydrous transition metal oxides are valuable materials as and intermediate layer. These materials are characterized by high electrical capacity and low transfer-charge resistance. Tested electrodes show high potential stability, close to Nernstian response, and wide linear range.

In the construction of carbon paste electrodes, the majority of pastes are based on graphite powder [21,23,26]. This paper presents electrodes with carbon paste based on CB and modified by hydrated transition metal oxides: (i) hydrous ruthenium dioxide and (ii) hydrous iridium dioxide. The combination of these materials is promising as a paste in the construction of ion-selective electrodes. Moreover, the tested paste with hydrous ruthenium dioxide has been re-modified by the addition of poly(3-octylthiophene-2,5-diyl) (POT) which, according to reports, has a positive effect on the properties of the intermediate layers of solid-contact electrodes [12,27,28]. The developed electrodes can be used to determine nitrate ions in liquid samples and soil samples. Controlling the content of nitrate ions in the soil is important from the point of view of plant growth.

## 2. Results and Discussion

### 2.1. Measurements

**Figure 1:**
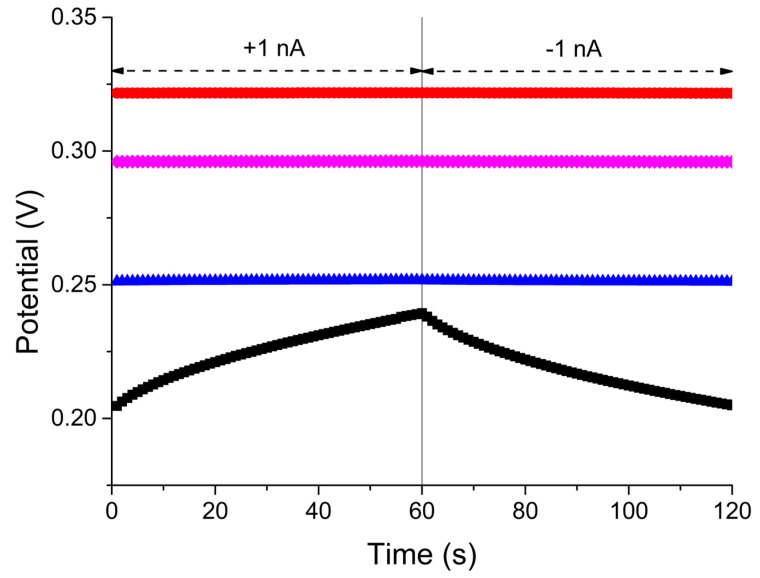
Chronopotentiogram for tested electrodes: “0” (black), “1” (red), “2” (blue), and “2a” (magenta) registered in 10^−3^ M KNO_3_.

In the first stage of research, each paste was characterized by chronopotentiometry (Figure 1). On the basis of these chronopotentiometry measurements, the electrical capacity, potential drift, and resistance were calculated. The obtained results are shown in Table 1. Each modification of the paste ”0” enlarged the electrical capacity of the paste. The highest value was obtained for the paste modified with hydrated ruthenium dioxide. The modification with hydrated transition metal oxides significantly increased the capacitance of the tested material. The addition of POT decreased capacity to a value lower than paste “2”, but was still higher than paste ”0”.

After determining the electrical parameters of the tested pastes, the electrodes were covered with ISM. Ready-to-use electrodes were tested again by the chronopotentiometry method. The measurement was carried out at a current of 10 nA. The results are summarized in Table 2.

### 2.2. Wettability

Hydrophobicity is an important parameter of materials used as an intermediate layer. High hydrophobicity prevents water penetration under the ionselective membrane and detachment of the membrane from the electrode surface. The hydrophobicity of the materials was tested by measuring the contact angle between a drop of water and the surface of the paste. In this test, 2 µL redistilled water drops was used. For subsequent pastes, the following contact angle values were obtained: 108° for “0”, 108° for “1”, 110° for “2”, and 118° for “2a”. All pastes are hydrophobic, which should positively affect the life of the electrodes. In addition, the addition of POT improves the hydrophobicity of the tested pastes.

### 2.3. Potentiometric Tests

For potentiometric tests, paste electrodes were coated with an ISM and conditioned in 10^−3^ KNO_3_ solution. The ready-to-use electrodes were connected to a potentiometer and measurements were taken in KNO_3_ standard solutions of concentration 10^−7^ to 10^−1^ M. In this way, the electrode characteristics were determined, including parameters such as standard potential and sensitivity.

#### 2.3.1. Potentiometric Response

**Figure 2:**
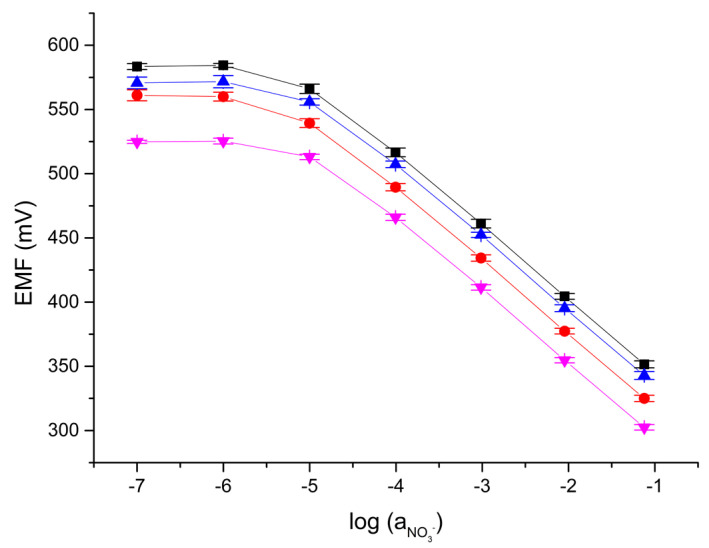
Potentiometric response of the tested electrodes: “0” (black), “1” (red), “2” (blue), and “2a” (magenta). Average value of potential after 24, 48, and 72 h of conditioning in 0.001 M KNO_3_, with standard deviation (n = 3).

The calibration curves for each type of electrode recorded after 24, 48, and 72 h of conditioning in a solution of 10^−3^ M KNO_3_ are presented in Figure 2. The standard deviations of the electrode parameters were determined on the basis of three calibrations performed on consecutive days for one electrode of each type. The obtained parameters are presented in Table 3.

All electrode slopes are close to the Nernstian value. The highest slope was obtained for electrode “0”. The most reproducible response was detected for electrode “2a”, with POT addition, which was confirmed by the lowest standard deviation of the normal potential. It confirms that the addition of POT improves reproducibility of normal potential. The lowest limit of detection was obtained for electrode “1” and the highest for electrode “2a”. However, the linear range of response is the same for each type of electrode.

The developed electrodes show a very stable response over time. Even after a long time of conditioning in 0.001 M *KNO_3_* (6 weeks), the electrodes still showed a linear response in the same range of nitrate activity. For example, the normal potential values of the “0” and “2a” electrodes, that were equal to 289.1 mV and 239.7 mV, respectively, after 3 days conditioning, changed of 2.4 and of 2.0 mV, respectively, after 6 weeks.

Electrode parameters of previously reported NO_3_^−^-selective electrodes were collected in Table 4. As can be observed, the results obtained in this work are comparable to those previously reported. It should also be taken into account that the tested electrodes are paste electrodes, which are different from glassy carbon (GC) disc electrodes with an intermediate layer and ISM. The sensitivity obtained for the tested electrodes is higher than for the electrodes with a layer of multi-walled carbon nanotubes (MWCNTs), but lower than for the electrodes with a layer of CB. The detection limit is comparable to that obtained for electrodes with a layer of chemically reduced graphene oxide (CRGO). In addition, the electric charge capacitance of all tested electrodes is higher than most of the previously presented solutions but lower than for electrodes with layers of CB Printex XE-2 and Vulcan XC-72. Moreover, presented paste electrodes are electrodes with a renewable surface. Once prepared, the electrode can be used for a long time with various membranes; it is sufficient to push out a little paste and level the surface. This does not require preparation and application of an intermediate layer each time.

**Figure 3:**
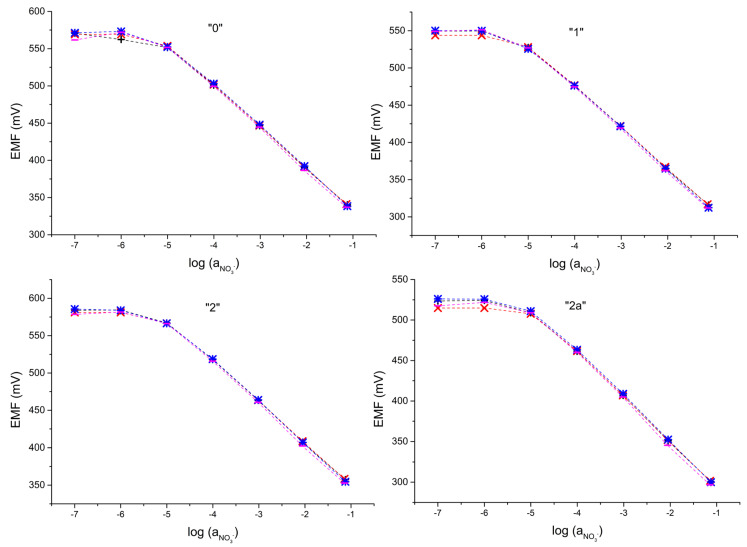
Potentiometric response of electrodes “0”, “1”, “2”, and “2a” recoded in several nitrate salts solutions: sodium nitrate (+), calcium nitrate (*), magnesium nitrate (x), and potassium (-).

In the next stage, the effect of the presence of various cations in the solution on the potentiometric response was examined, as shown in Figure 3. The response was tested in the presence of sodium, calcium, and magnesium ions. For all tested electrodes, no significant influence of the cation on the potentiometric response was observed. On this basis, it can be inferred that all tested electrodes can be successfully used for determinations in solutions with multiple cations.

#### 2.3.2. Selectivity

**Figure 4:**
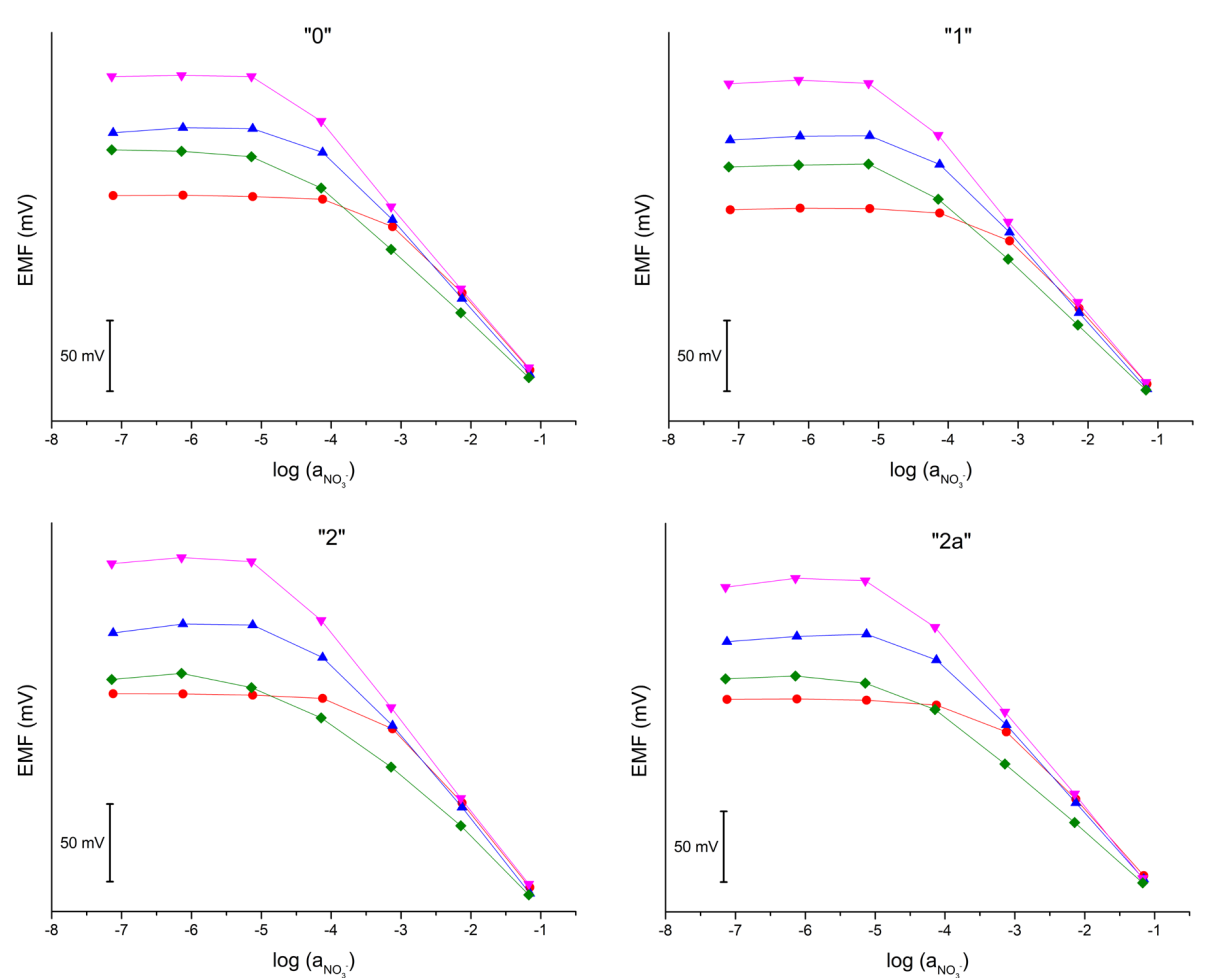
Potentiometric response of electrodes “0”, “1”, “2”, and “2a” recoded in solutions with addition several salts: 0.1 M potassium chloride (red), 0.1 M sodium acetate (blue), 0.05 M potassium sulfate (magenta), and 0.05 M dipotassium phosphate (green).

The influence of some common cations on the potentiometric response was also investigated. The study was carried out using the Fixed Interference Method. Sets of solutions with a constant concentration of the interfering ion and a variable concentration of the main ion were prepared. The following interfering ion additions were used: 0.1 M KCl, 0.05 M K_2_SO_4_, 0.1 M CH_3_COONa, and 0.05 M K_2_HPO_4_. A calibration curve was then recorded for each set of solutions. The obtained results are shown in the Figure 4. Based on the obtained results, activity coefficients were determined for each of the electrodes and collected in Table 5.

The results obtained for the subsequent electrodes are consistent, which proves that the selectivity is not dependent on the paste used, but rather on the ion-selective membrane. The worst selectivity was obtained for chloride ions, and the best for sulfate ions. 

#### 2.3.3. Stability and Reversibility of Response

**Figure 5:**
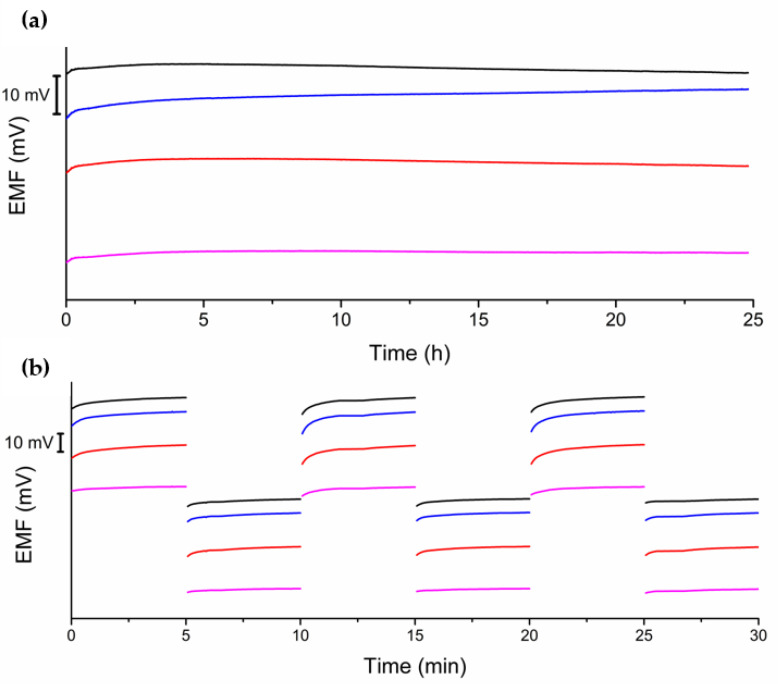
Stability test carried out in 10^−3^ M KNO_3_ (**a**) and reversibility test (**b**) of investigated electrodes “0” (black), “1” (red), “2” (blue), and “2a” (magenta) carried out in 10^−3^ and 10^−2^ M KNO_3_.

The next test conducted was the potential stability test. During a 25 h measurement, a signal was recorded in a 10^−3^ M NO_3_^−^ ion solution (Figure 5a). On the basis of the obtained results, the potential drift over time was determined. For the subsequent electrodes, the potential drift was equal to 0.12 mV/h for electrode “0”, 0.11 mV/h for electrodes “1” and “2”, and 0.02 mV/h for electrode “2a”. The addition of transitional metal oxides did not result in noticeable changes in the recorded potential drift. The use of materials with high electric charge capacity allowed for obtaining electrodes with a low potential drift over time. On the basis of the obtained results, it can be concluded that material modification by POT can significantly reduce the potential drift over time.

In the next stage, a potential reversibility test was performed. During the measurement, the potential was recorded alternately in solutions with a concentration of 10^−3^ and 10^−2^ M NO_3_^−^ ions. Each measurement in the subsequent solution lasted 5 min (Figure 5b). On the basis of the obtained data, it is possible to calculate the standard deviation for each measurement in the same solution, which allows comparison of the potential reversibility of different types of electrodes. Additionally, the response time of the electrodes can be determined, which is the time after which the potential value reaches 95% of the equilibrium state value. The response time for all electrodes is short and is only a few seconds. The standard deviation in the 10^−3^ M NO_3_^−^ ion solution was 0.1 mV for electrodes “0”, “1”, and “2a”, and 0.02 mV for electrode “2”. In the 10^−2^ M NO_3_^−^ ion solution, it was 0.2 mV for electrodes “0”, “1”, and “2”, and 0.01 mV for electrode “2a”.

#### 2.3.4. pH and Light Sensitivity

**Figure 6:**
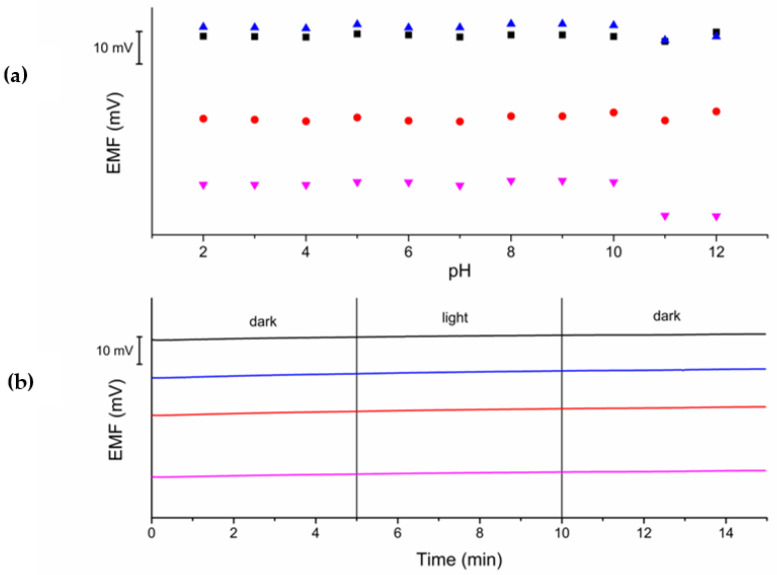
pH sensitivity (**a**) and light sensitivity (**b**) tests of the investigated electrodes “0” (black), “1” (red), “2” (blue), and “2a” (magenta) carried out in 10^−3^ M KNO_3_.

In the pH sensitivity test, the potential was measured sequentially in a series of KNO_3_ solutions with pH ranging from 2 to 12 at a concentration of 10^−3^ M KNO_3_ (Figure 6a). The pH of the solutions was adjusted using 1M solutions of HCl and NaOH. Above a pH value of 10, the potential obtained in the measurement is lower than the results obtained for lower values of pH. It does not depend on the paste used, but rather on the PVC membrane properties. The range of insensitivity for the response depending on pH for all tested electrodes is 2–10. Therefore, these electrodes can be used successfully in the given range and obtain an undisturbed response.

In the light sensitivity test, the potential was measured in a 10^−3^ M KNO_3_ solution for 5 min in the dark, followed by 5 min in the daylight and 5 min in the dark again (Figure 6b). No visible changes were observed in the electrode response. The test showed that electrode exposure to light does not affect the response of the tested electrodes, and that they can be used in both daylight and darkness conditions, which gives a wide field of application in various conditions.

#### 2.3.5. Water Layer Test

**Figure 7:**
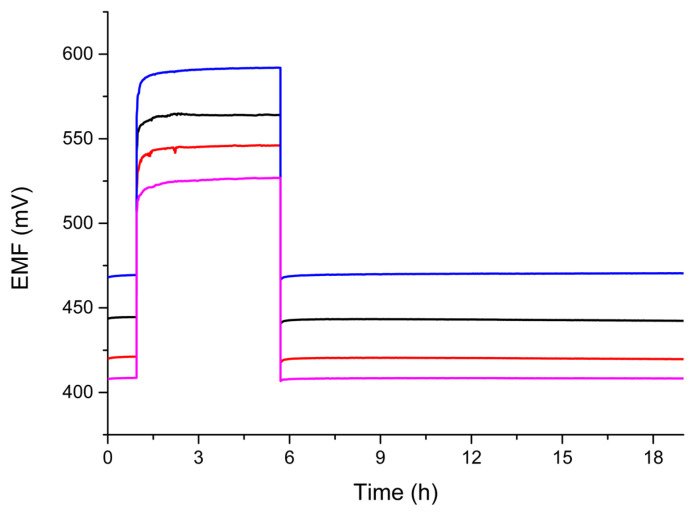
Water layer test of investigated electrodes “0” (black), “1” (red), “2” (blue), and “2a” (magenta) carried out for 1 h in 10^–3^ M KNO_3_ solution, then for 5 h in 10^−3^ M KCl solution, and then 13 h in 10^–3^ M KNO^3^ solution.

The water layer test is used to determine whether a water layer forms at the interface of the ISM phase and the paste/transition layer. Its presence can negatively affect the stability of the electrode response and cause membrane delamination on the electrode surface. In the conducted test, the potential was measured alternately in the main ion solution and in the interferent solution [32]. The measurement was carried out for 1 h in 10^−3^ M KNO_3_ solution, then for about 5 h in 10^−3^ M KCl solution, and again in the main ion solution for approximately 13 h (Figure 7). During the measurement, the response of the tested electrodes stabilised both in the interferent solution and upon return to the measurement in the main ion solution. The potential drift characteristic is not visible on the graphs after returning to the measurement in the main ion solution, indicating the absence of a water layer at the interface. The use of hydrophobic layers successfully prevented water from penetrating under the membrane, thus ensuring good potential stability and preventing the detachment of the ISM from the electrode surface.

#### 2.3.6. Analytical Applications

In order to demonstrate the feasibility of analyses with the designed paste electrodes in environmental analysis, two electrodes, “2” and “2a”, were used to measure the concentration of nitrate ions in soil samples. The electrode “2a” was chosen because it had the best stability and reversibility of response, and electrode “2” as an unmodified version of electrode “2a”. Soil samples were collected from agricultural fields. 100 g of each soil was weighed and 20 mL of deionised water was poured over it. On the next day, the measurement was carried out directly in the soils collected by direct potentiometry. A calibration solution of 0.05 M K_2_SO_4_ was added to ensure constant ionic strength. The analysis was performed using the calibration curve method. The results of the analysis are presented in Table 6.

## 3. Materials and Methods

### 3.1. Chemicals

The components of the carbon paste were paraffin oil (Sigma-Aldrich, Burlington, MA, USA), carbon black (3D Nano, Krakow, Poland), ruthenium (IV) oxide hydrate (Alfa Aesar, Lancashire, UK), iridium (IV) oxide dihydrate (Alfa Aesar), and poly(3-octylthiophene-2,5-diyl) (Sigma-Aldrich).

The ion-selective membrane components—nitrate ionophore V, lipophilic salt-tridodecylmethylammonium chloride (TDMACl), 2-nitrophenyl octyl ether (o-NPOE), and poly(vinyl chloride) (PVC)—were dissolved in tetrahydrofuran. All components were purchased from Sigma-Aldrich. 

Potassium nitrate (KNO_3_) and other chemicals were purchased from POCH (Gliwice, Poland), and solutions of NO_3_^−^ ions with concentrations of 10^−7^ to 10^−1^ M were used for potentiometry, chronopotentiometry, and EIS measurements.

### 3.2. Electrode Preparation

Base paste for the electrode marked as “0”, with the composition of 0.4 g of carbon black (CB) and 0.3 g of paraffin oil, was ground in a mortar until a homogeneous mixture was obtained. Then, for modification, two samples of 0.1 g of base paste were mixed with 0.025 g of hydrated ruthenium oxide for electrode (CB + RuO_2_·2H_2_O) “1” or hydrated iridium oxide (CB + IrO_2_·H_2_O) for electrode “2”. To modify the pastes obtained in this way with the addition of POT, 0.05 g of the paste with ruthenium dioxide hydrate was mixed with 0.01 g of POT (CB + RuO_2_·2H_2_O + POT)—electrode “2a”.

The pastes prepared in the described procedure were used to fill the cavity of the paste electrode with stainless steel rod. The cavity diameter was equal to 3 mm. The prepared electrodes were subjected to electrochemical measurements: cyclic voltammetry and chronopotentiometry. At this stage, the hydrophobicity of the tested materials was also measured.

After electrochemical characterization, the electrode surface was covered with 70 µL ISM solution using drop-casting method. The ISM was prepared according to a slightly modified procedure described by Watts et al. [33]. The ISM solution had the following composition: nitrate ionophore V 1.10% (*w*/*w*), TDMACl 0.70% (*w*/*w*), o-NPOE 65.00% (*w*/*w*), and PVC 33.20% (*w*/*w*). All components of the membrane of total weight 0.126 g were dissolved in 1 mL of THF. Before measurements, the membrane-covered electrodes were conditioned in 10^−3^ M KNO_3_ solution for 3 days of calibration and then stored dry and conditioned for at least an hour before the measurement. Ready-to-use electrodes have again been subjected to the tests mentioned and have been widely characterized potentiometrically.

### 3.3. Measurements

Chronopotentiometry was used to develop electrochemical parameters of the tested pastes. The measurements were performed using the Autolab General Purpose Electrochemical System (AUT302N.FRA-2-AUTOLAB, Eco Chemie, Utrecht, The Netherlands) that cooperated with NOVA 2.1.4 software. Measurements were carried out in a three-electrode system with the single-junction Ag/AgCl and 3 M KCl reference electrode (catalog number 6.0733.100 Ω Metrohm, Herisau, Switzerland), a carbon glass rod as auxiliary electrode and one of the tested electrodes as an indicator electrode.

Chronopotentiograms were obtained according to the method proposed by Bobacka [34]. The potential of the electrodes was registered during the forced current flow through the system. The direction of current flow was changed after 60 s of measurements. This allowed the calculation of three important parameters: electrical capacity (C = I(∆t/∆Edc)), resistance (R = E/I), and potential drift (∆Edc/∆t). All electrical characteristic measurements were carried out in a 10^−1^ M KCl solution. 

Potentiometric measurements were made using a 16-channel EMF meter (Lawson Labs, Inc., Malvern, PA, USA) connected with a single-junction Ag/AgCl/KCl (3 M solution) reference electrode (catalog number 6.0733.100 Ω Metrohm, Herisau, Switzerland) or Radiometer REF201 (catalog number 12003-F12, Radiometer Analytical, Lyon, France), a platinum auxiliary electrode and a tested electrode. The potentiometric response was recorded in standard KNO_3_ solutions of concentrations ranging from 10^−1^ to 10^−7^ M.

## 4. Conclusions

The effect of the application of various pastes based on carbon black in paste ion-selective electrodes was studied in this work. Prepared pastes contained carbon black and hydrous transition metal oxides—ruthenium and iridium. For pastes with the addition of both metal oxides, a positive effect on the paste charge capacity was observed in relation to the reference paste containing only carbon black. The paste with hydrous ruthenium dioxide, characterized by the best capacitance (470 µF), was additionally modified by poly(3-octylthiophene-2,5-diyl). This modification results in the PVC-based electrode with the highest capacitance (130 µF), which proves the positive effect of polymer modification.

All tested electrodes show a slope of calibration curve close to the Nernstian value. Both the addition of metal oxides and following modification with polymer has no significant impact on the limit of detection compared to reference paste. The lowest limit of detection was calculated for the electrode with paste with hydrous iridium oxide (10^−5.32^). Modification with poly(3-octylthiophene-2,5-diyl) produced the electrodes with the best stability of potential (0.02 mV/h) and reversibility of response (0.01 mV). All investigated electrodes are insensitive to light condition and pH condition in range 2–10. Moreover, two electrodes were used to determine nitrate ions directly in soil samples, giving comparable results. Satisfactory parameters and insensitivity to the presence of different cations in sample give the opportunity to construct multi sensors based on paste electrodes.

## Figures and Tables

**Table 1:** Electrical parameters calculated on the basis of chronopotentiometric measurements.

Paste	Paste Number	Potential Drift µV/s	Capacitance µF	Resistance kΩ
CB	0	407 ± 22	2.40 ± 0.15	652 ± 7
CB + IrO_2_·H_2_O	1	3.8 ± 0.2	263 ± 15	27.5 ± 0.6
CB + RuO_2_·2H_2_O	2	2.3 ± 0.5	470 ± 13	30.5 ± 0.8
CB + RuO_2_·2H_2_O + POT	2a	3.8 ± 0.5	317 ± 15	10 ± 0.8

**Table 2:** Electrical parameters of ready-to-use electrodes calculated on the basis of chronopotentiometric measurements.

Electrode	Potential Drift µV/s	Capacitance µF	Resistance kΩ
“0”	146 ± 2	0.83 ± 0.04	270.3 ± 0.5
“1”	117 ± 9	86 ± 6	348.7 ± 0.4
“2”	181 ± 15	98 ± 5	607.1 ± 0.1
“2a”	116 ± 15	130 ± 2	237.9 ± 0.2

**Table 3:** Calibration curve parameters of the studied ISEs after 24, 48, and 72 h of conditioning.

Electrode	Normal Potential mV	Slope mV/dec	Limit of Detection M	Linear Range M
“0”	287.3 ± 2.1	−57.4 ± 0.4	10^−5.27 ± 0.06^	10^−5^–10^−1^
“1”	260.8 ± 2.1	−57.2 ± 0.2	10^−5.32 ± 0.08^	10^−5^–10^−1^
“2”	278.6 ± 3.2	−57.2 ± 0.2	10^−5.22 ± 0.05^	10^−5^–10^−1^
“2a”	238.7 ± 1.9	−56.9 ± 0.1	10^−5.15 ± 0.05^	10^−5^–10^−1^

**Table 4:** Characteristic of previous reported NO_3_^−^-selective electrodes.

Electrode	Normal Potential mV	Slope mV/dec	Limit of Detection M	Linear Range M	Capacitance µF	Ref.
GC/MWCNT/NO_3_^−^-ISM	-	−55.1	2.8 × 10^−8^	10^−7.09^–10^−2^	49.2	[29]
GC/CRGO/NO_3_^−^-ISM	-	−57.9	3 × 10^−5^	10^−4.3^–10^−1^	-	[30]
GC/CB(Printex XE-2)/NO_3_^−^-ISM	191.1 ± 1.1	−58.96	1.26 × 10^−6^	10^−6^–10^−1^	289	[31]
GC/CB(Vulcan XC-72)/NO_3_^−^-ISM	189.6 ± 0.2	−58.6	2.51 × 10^−7^	10^−6^–10^−1^	203	[31]
GC/CB(Printex U)/NO_3_^−^-ISM	190.9 ± 2.0	−59.42	3.16 × 10^−7^	10^−6^–10^−1^	28	[31]
GC/TTF/NO_3_^−^-ISM	36.7 ± 0.5	−58.85	2.5 × 10^−6^	10^−5^–10^−1^	5.99	[20]

**Table 5:** Selectivity coefficients calculated for NO_3_^−^-selective electrodes.

Electrode	Selectivity Coefficient $\log K_{NO_{3}^{-},j}$			
	Cl^−^	CH_3_COO^−^	SO_4_^2−^	HPO_4_^2−^
“0”	−2.3	−3.3	−3.8	−3.7
“1”	−2.4	−3.4	−3.9	−3.8
“2”	−2.3	−3.3	−3.9	−3.7
“2a”	−2.3	−3.3	−3.8	−3.7

**Table 6:** Results of determination of nitrate ions in soil samples.

Sample	Nitrate Concentration [g/L]	
	“2”	“2a”
Unfertilized soil	<LoD	<LoD
Chemically fertilized soil	1.40 ± 0.02	1.40 ± 0.01
Soil fertilized with chicken manure	0.142 ± 0.003	0.134 ± 0.002

## Data Availability

Not applicable.
